# Supplementary material for: Elastic Alfven waves in elastic turbulence
Source: Nat Commun. 2019 Feb 8;10:652. doi: 10.1038/s41467-019-08551-0 (PMC6368571; doi:10.1038/s41467-019-08551-0)
Supplement: Supplementary file 1 — Supplementary Information [file 41467_2019_8551_MOESM1_ESM.pdf]

Elastic Alfven waves in elastic turbulence

Varshney et al.

Supplementary Figures

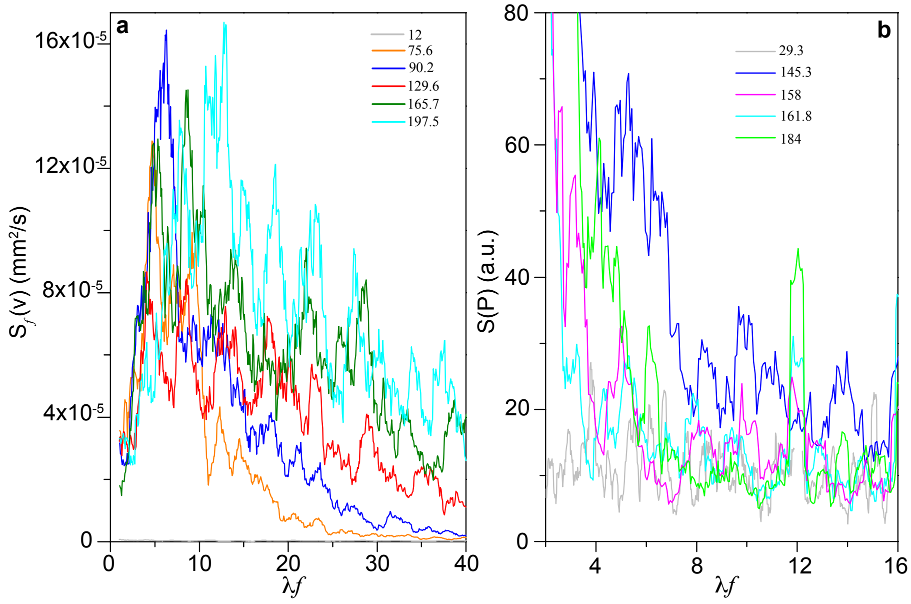

Supplementary Figure 1. **Cross-stream velocity and pressure spectra.** (**a**) Cross-stream velocity power spectra $S_f(v)$ versus the normalized frequency λf in linear coordinates to emphasize the oscillation peaks. The spectra are obtained at $(x/R, y/R)$=$(5.2, 0.56)$ and for different Wi. The oscillation peaks are also observed in the spectra of absolute pressure fluctuations in linear coordinates, as shown in (**b**) for different Wi.

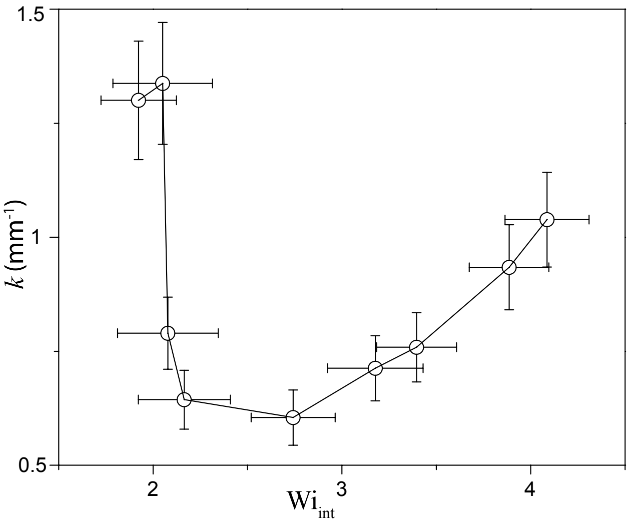

Supplementary Figure 2. **Dependence of** k **of elastic waves on** $\mathrm{Wi}_{\mathrm{int}}$. Estimated wave number of the elastic waves ($k = 2\pi f_{\mathrm{p}}/c_{\mathrm{el}}$) as a function of $\mathrm{Wi}_{\mathrm{int}}$. The total error on k is estimated based on errors of f_{p} and c_{el}, and for $\mathrm{Wi}_{\mathrm{int}}$ the error bars are calculated based on the standard deviation from the mean value of $(\partial u/\partial y)$.

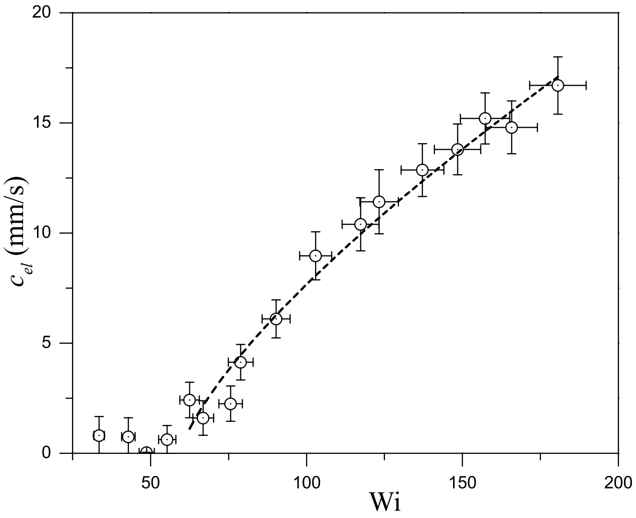

Supplementary Figure 3. **Elastic wave speed versus** Wi. Dependence of elastic wave speed c_{el} on Wi, where the dashed line is a fit to the data of the form $c_{\mathrm{el}} \sim (\mathrm{Wi} - \mathrm{Wi}_{\mathrm{c}})^{0.73\pm0.12}$ that yields $\mathrm{Wi}_{\mathrm{c}} = 59.7 \pm 1.8$. The error on c_{el} is estimated based on the s.d. of the linear fit of Δx versus τ_{p}, and for Wi they are calculated based on the standard deviation from the mean value of fluid discharge rate Q.

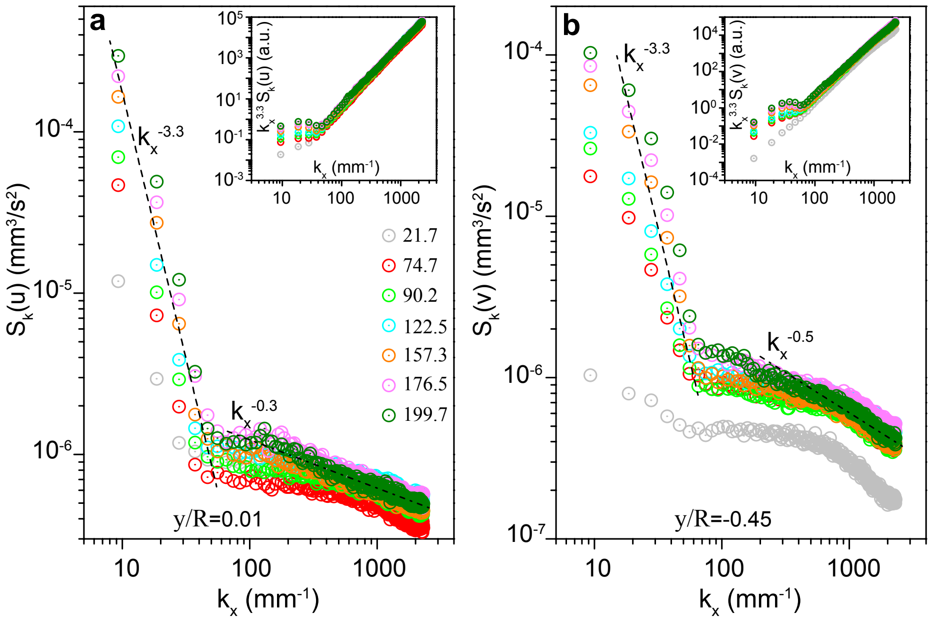

Supplementary Figure 4. **Spatial power spectra of** u **and** v**.** Spatial power spectra of (**a**) streamwise velocity $S_\mathrm{k}(u)$ and (**b**) cross-stream velocity $S_\mathrm{k}(v)$, for different values of Wi. The dashed lines are fits to the steep power-law decays for both $S_\mathrm{k}(u)$ and $S_\mathrm{k}(v)$ spectra at low k_x. The dash-dotted lines are fits to the flat power-law decays at high k_x. Insets in (**a**) and (**b**) show the respective compensated plots, i.e. $k_x^{3.3} S_\mathrm{k}(u)$ and $k_x^{3.3} S_\mathrm{k}(v)$, with k_x.
