# Supplementary material for: Elastic Alfven waves in elastic turbulence
Source: Nat Commun. 2019 Feb 8;10:652. doi: 10.1038/s41467-019-08551-0 (PMC6368571; doi:10.1038/s41467-019-08551-0)
Supplement: Supplementary file 3 — Description of Additional Supplementary Files [file 41467_2019_8551_MOESM3_ESM.pdf]

Description of Additional Supplementary Files

Supplementary movies of polymer solution flow, both inside and outside the obstacles' region, are recorded at 1 fps with an exposure time 500 ms and displayed at 10 fps. White arrows indicate the flow direction.

Supplementary Movie 1:

The movie shows flow dynamics in the elastic turbulence (ET) regime at Reynolds number Re=0.009 and Weissenberg number Wi=103.

Supplementary Movie 2:

The movie shows flow dynamics in the ET regime at Re=0.013 and Wi=148.4.

Supplementary Movie 3:

The movie shows flow dynamics in the ET regime at Re=0.017 and Wi=197.5.
